# Distal Pullout Strengths of the Biceps Long Head Tendon for Different Adjacent Tissue and Tendon Pathologies during Rotator Cuff Repair

**DOI:** 10.1155/2018/4267163

**Published:** 2018-09-09

**Authors:** Young Jun Kim, Ohhyo Kwon, Hwa-Ryeong Lee, Sae Hoon Kim

**Affiliations:** Department of Orthopaedic Surgery, Seoul National University College of Medicine, Seoul National University Hospital, Republic of Korea

## Abstract

*Purpose. *Pathologies of the long head of the biceps tendon (LHBT) are frequently recognized in cases of rotator cuff tear. Recommendations for managing such pathologies remain debatable, and distal migration of tenotomized biceps is always a concern when only tenotomy is performed.* Methods. *Seventy patients of mean age 60.4 ± 6.9 years (range: 44 to 82 years) were included in this retrospective study. During subpectoral tenodesis in rotator cuff repair, pullout tensions were measured using a digital tensiometer. Measured tensions obtained were analyzed with respect to sex, tear involvement of the subscapularis, and the presence of a partial tear of LHBT, type II SLAP lesion, subluxation/dislocation of the biceps, or a pulley lesion.* Results*. Mean LHBT pullout tension for the 70 study subjects was 86.5 ± 42.1 N (26.7-240.5 N). Distal LHBT pullout tension was significantly greater for men than women (93.2 ± 42.7 N versus 73.7 ± 38.7 N, P = 0.041). However, LHBT pullout tensions were not significantly associated with different pathologies of surrounding tissues or of LHBTs (all Ps > 0.05).* Conclusion. *The study failed to show pullout tension differences associated with pathologies affect distal migration of a tenotomized LHBT. Gender was the only factor found to affect LHBT pullout strength. Risk of distal migration of tenotomized LHBT could not be predicted with intraoperative arthroscopic pathologic findings.

## 1. Introduction

Lesions of the long head of the biceps tendon (LHBT) and surrounding tissues, such as partial tear of the LHBT, subluxation or dislocation of LHBT, a superior labral anterior and posterior lesion (SLAP lesion), and tear of the anterior or posterior biceps pulley (pulley lesions), are easily observed during rotator cuff repair [1, 2]. Various procedures have been shown to be effective for the treatment of LHBT during rotator cuff repair [3–5], but no consensus has been reached regarding the most effective treatment.

Biceps tenotomy is a recognized, successful procedure [6, 7], but there are always concerns of Popeye deformity or cramping pain and strength loss due to distal migration of the tendon, and thus, tenodesis of the LHBT has been recommended by some authors [8]. However, not all patients that have undergone biceps tenotomy later experience Popeye deformity. In fact, the reported rate of Popeye deformity is between 3% and 63% [8–12]. Pathologies of the LHBT and surrounding tissues are known to be critical for LHBT stability, and it is possible that the vinculum prevents distal migration of the LHBT. The vinculum is usually described to be within the flexor digitorum profundus and is important for providing nutrition to the LHBT [13, 14], and, for preventing proximal migration of the flexor tendon after tendon rupture [13], the vinculum of LHBT could resist distal pull of the tendon because it has a constant structure in the LHBT [15]. In one study [15], the pullout strength of the LHBT after tenotomy was measured in a human cadaver, but this study did not represent clinical situations with associated pathologies. Therefore, information on the pullout strength of the LHBT in real clinical situations is valuable because it enables the risk of distal migration of the tendon to be predicted in the presence of different pathologies.

Subpectoral tenodesis of the LHBT has recently gained in popularity because it could address “hidden lesions” of LHBT in the bicipital groove as well as providing satisfactory outcomes [16–19]. This procedure has been continuously performed in our institution since Apr 2015, and, during this procedure, LHBT is pulled out from the bicipital groove and pullout strength measured.

In this study, we analyzed distal pullout tensions of LHBTs in the presence of different pathologies of the LHBT and surrounding tissues. To the best of our knowledge, this is the first study to analyze LHBT pullout strengths according to pathologies of the LHBT and of adjacent tissues in actual clinical situations. We hypothesized that the presence of these pathologies would influence LHBT pullout tensions.

## 2. Materials and Methods

### 2.1. Inclusion and Exclusion Criteria

Patients were enrolled from April 2015 to June 2016. The following inclusion criteria were applied: (1) full-thickness rotator cuff tear verified by preoperative MRI at time of surgery, (2) biceps tenodesis performed due to a LHBT lesion or due to a pathology of surrounding tissue (partial tear of the LHBT, subluxation or dislocation of the LHBT, type II SLAP lesion, or pulley lesions), and (3) pullout tension of the LHBT measured during subpectoral tenodesis. The decision for tenodesis was mainly based on arthroscopic findings. Exclusion criteria were revision surgery of rotator cuff tear, partial-thickness rotator cuff tear, and complete LHBT rupture. Finally, 70 patients were enrolled. All protocols were approved by the Institutional Review Board of our institution (IRB no. H-1603-137-751).

### 2.2. Surgical Procedures and Tension Measurement

**Figure 1:**
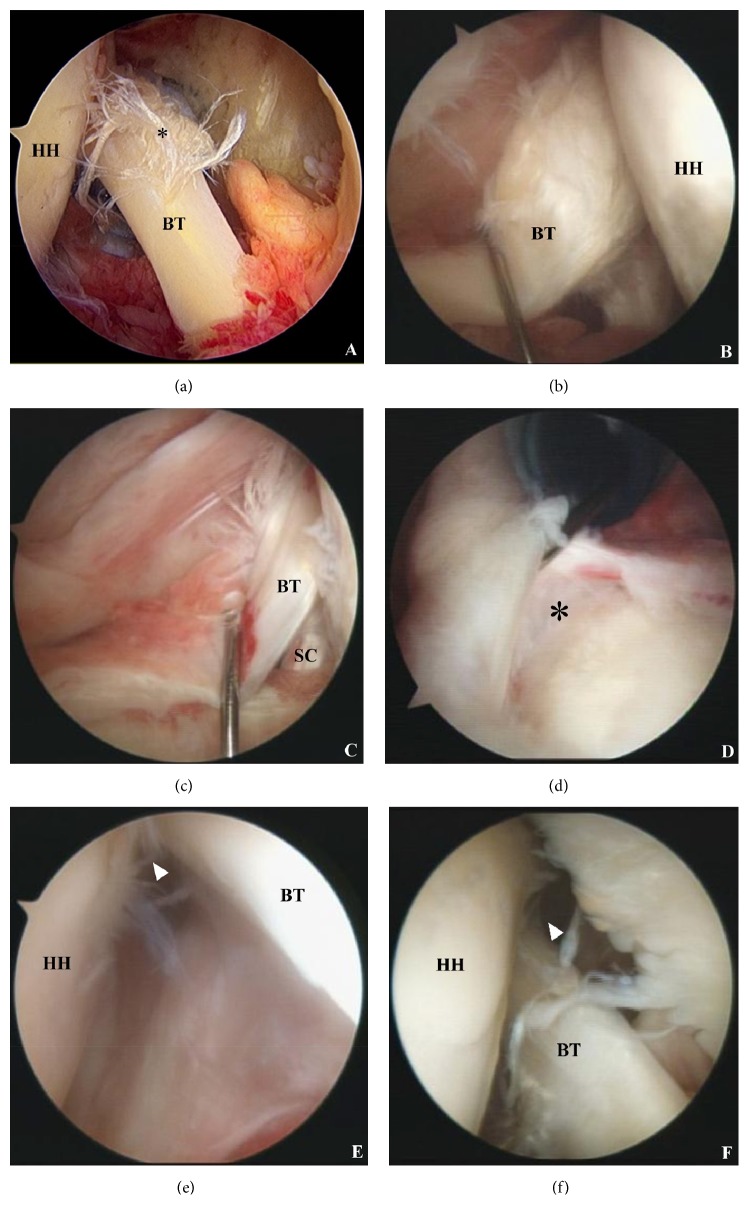
(a) Partial tear of the long head of the biceps (asterisk), (b) partial tear of the long head of the biceps with hypertrophy, (c) intraarticular subluxation of the biceps tendon medial to the subscapularis, (d) type II SLAP lesion (asterisk), (e) anterior pulley lesion (arrowhead), and (f) posterior pulley lesion (arrowhead); BT: biceps tendon, HH: humeral head, and SC: subscapularis.

All surgical procedures were conducted by a single surgeon (K.S.H.). With a patient in the lateral decubitus position, a scope was introduced to the intra-articular space and intra-articular pathologies were addressed; in particular, LHBT lesions, biceps pulley lesions, and subscapularis tear were carefully evaluated. When a decision to perform biceps tenodesis was made, the LHBT was tagged with a PDS suture and released from the glenohumeral joint. The open subpectoral tenodesis technique was utilized in all patients requiring biceps tenodesis due to a partial tear (> 50%) of the LHBT (Figures 1(a) and 1(b)), LHBT subluxation or dislocation (Figure 1(c)), type II SLAP lesion (Figure 1(d)), or pulley lesions (Figures 1(e) and 1(f)) during the index period.

**Figure 2:**
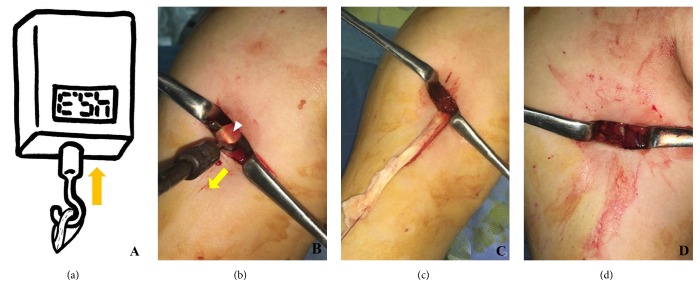
(a) Schematic figure and photograph of pullout strength measurement of the long head of the biceps tendon. (b) The tendon was attached to the hook of the tensiometer and maximal tension required pull the tendon from the bicipital groove was measured. (c) The long head of the biceps tendon was pulled out of the bicipital groove. (d) The long head of the biceps tendon was fixed using a suture anchor and the remnant biceps tendon was excised.

The surgical procedure is conducted as follows. A longitudinal incision (~2-3 cm in length) is made at the biceps centered at the inferior margin of the pectoralis major tendon. Location of the incision is verified by muscle movement by pulling the previously placed LHBT tagging suture. After blunt dissection of the brachial fascia, the LHBT is easily found by superolateral retraction of the pectoralis major tendon sling. Pullout tension of LHBT is then measured with a digitalized tensiometer (FGN-50, Nidec-Shimpo Co., Japan) prepared in a sterile fashion. The LHBT is loaded at the hook of the tensiometer (Figures 2(a) and 2(b)). The tensiometer was set to measure peak tension and manual traction was applied to the tensiometer until LHBTs were pulled out of bicipital grooves (Figure 2(c)).

The LHBT was fixed using a soft anchor (JuggerKnot; Biomet, Warsaw, IN) and sutured using the LHBT using a lasso-loop stitch (Figure 2(d)) [21]. Drilling and anchor insertion were performed after placing a guide directly underneath the pectoralis tendon at the tenodesis site. To maintain the length-tension relationship of the LHBT, it was sutured at the musculotendinous junction with a lasso-loop stitch and tied firmly [22].

After finishing subpectoral tenodesis of LHBT, the scope was moved to the subacromial space and standardized rotator cuff repair was performed. Full-thickness rotator cuff tears were repaired using an all-arthroscopic, single row repair technique.

### 2.3. Statistical Analyses

Statistical analysis was performed using the SPSS software package (version 21.0, IBM SPSS statistics, Chicago, IL), and Statistical significance was accepted for p values < 0.05. Descriptive analysis, correlation analysis, Student's* t* test, the Mann–Whitney* U* test, analysis of variance (ANOVA), or the Kruskal-Wallis test was used, as appropriate.

## 3. Results

For all study subjects, mean LHBT pullout tension was 86.5 ± 42.1 N (26.7-240.5 N). According to the tear size classification of DeOrio and Cofiled [23], there were 4 (5.7%) small, 34 (48.6%) medium, 13 (18.6%) large, and 19 (27.1%) massive tears. Subscapularis involvement was observed in 54 cases (77.1%), and, according to the classification of Laffose et al. [20], there were 26 type I, 23 type II, and 5 type III subscapularis tears. Patient data is listed in Table 1.

Distal LHBT pullout tension was statistically higher in men than women (m: 93.2 ± 42.7 N versus f: 73.7 ± 38.7 N, P = 0.041). However, in total cohort, age, height, and weight were not related to the pullout tension by correlation analysis (R = 0.043 and P = 0.725; R = 0.147 and P = 0.225; and R = 0.187 and 0.122, respectively) and subgroup analysis in male and female showed biceps pullout tension was not related to height and weight of the patient (for male height: R = -0.48 and P = 0.749, and weight: R = 0.153 and P = 0.309, and for female height: R = -0.54 and P = 0.802, and weight: R = -0.111 and P = 0.605)

For pathology-based analysis, pullout tensions were not different in the presence of different LHBT pathologies (normal: 77.0 ± 39.0 N; partial tear: 86.5 ± 34.5 N; partial tear with hypertrophy: 93.8 ± 53.3 N, P = 0.440). Furthermore, neither the presence of biceps hypertrophy (no: 83.1 ± 36.0 N versus yes: 93.8 ±53.3 N, P = 0.595) nor a subscapularis tear (no tear: 83.6 ± 47.8 N versus tear: 87.3 ± 40.7 N, P = 0.576) affected pullout strength. Similarly, subscapularis tear severity (type I: 94.0 ± 44.0 N; type II: 82.1 ± 39.1 N; type III: 77.1 ± 29.1 N, P = 0.540), presence of a SLAP lesion (no: 83.8 ± 44.7 versus yes: 90.5 ± 38.3, P = 0.281), and LHBT location (subluxation/dislocation: 85.5 ± 42.0 versus normal: 87.4 ± 42.8, P = 0.796) did not affect pullout strength. Only ten patients had a normal biceps pulley or an anterior pulley lesion only (5 cases each, Table 1). However, the analysis showed that pullout tension was unaffected by the presence of a pulley lesion (no involvement: 90.5 ± 44.7 N versus anterior pulley involvement: 83.3 ± 40.2 N, P = 0.460). Since the gender showed statistical significance in our variables, subgroup analysis had been performed in men and women for above pathologies that there was no statistical difference in the pullout tension had been found for each variable (Tables 2 and 3).

## 4. Discussion

The main finding of this study is that pathologies LHBT lesions and of adjacent tissues do not affect distal pullout strength of the LHBT. In fact, the only factor found to significantly affect pullout tension was gender (men had a higher mean pullout tension). In subgroup analysis for male and female patients, it is also shown that there is no statistical significance in the pullout tension according to different pathologies. However, relevant numbers in some cases were small, especially in female population (i.e., biceps tear and subscapularis tear); therefore, statistical result cannot be made for especially subscapularis tear in female patients.

For all 70 study subjects, a mean pullout tension of 86.5 ± 42.1 N (range; 26.7-240.5 N) was needed to pull the LHBT out of the bicipital groove. Given that a maximum force of 55 N was found to be generated in the LHBT by contraction of the biceps brachii in a physiologic cross-sectional area study [24, 25], mean LHBT pullout tension as determined in the present study is considerably higher. Furthermore, we found that 17 (24.3%) patients had a pullout tension of < 55 N, which is markedly lower than the 102.7 ± 76.0 N (range; 17.4-227.6 N) found in a cadaver study [15].

The result that gender was meaningfully affected LHBT pullout tension is in accordance with that of Lim et al. [12], who reported that the development of Popeye deformity was not significantly influenced by age, body mass index, or arm dominance and concluded that the only predisposing factor was a male gender. Various other lesions that might have affected distal migration of a tenotomized LHBT were also included in the present study. It has been previously suggested hypertrophy of the LHBT, the so-called “hourglass biceps”, may resist distal migration of a tenotomized LHBT because it causes the tendon to become “stuck” in the groove [26, 27]. Ahmad et al. [27] reported diseased biceps tendons exhibiting hypertrophy or flattening increase the force required to travel through the bicipital groove, but this was not observed in the present study. In another study, the risk of Popeye deformity was found to be influenced by the coexistence of supraspinatus and subscapularis tears [28]. However, in the present study LHBT pullout tension was not significantly influenced by the presence of or the severity of a subscapularis tear.

Numerous studies have compared the outcomes of biceps tenotomy and tenodesis. However, even, in young patients, no outcome differences were observed [29]. Nevertheless, some authors recommend biceps tenodesis for decreasing the risk of Popeye deformity and for maintaining length-tension relationships [22, 30]. Several different tenodesis techniques related to fixation method (interference screw, suture anchor, or sutureless ancho), position of tenodesis (suprapectoral and subpectoral), and open versus arthroscopic tenodesis have been described [31]. Recently, subpectoral tenodesis was reported to produce good results and recommended for the treatment of “hidden” and extra-articular lesions [16]. Our procedure involves pulling out the LHBT below the pectoralis sling, and, thus, we had the opportunity of measuring LHBT pullout strengths while performing subpectoral tenodesis.

Based on our findings, tenotomy could be a good option in rotator cuff tear patients when LHBT pullout strength is above maximum physiologic biceps muscle strength in the majority of patients, and, in those patients, Popeye deformity may not develop. Reported failure strengths of suture anchor fixation of the LHBT range from 99.1 N to 287.7 N [32–36], and, in the present study, LHBT pullout strength was comparable to suture anchor fixation strength at failure. Therefore, there should be structure which resists distal migration of LHBT and it is thought to be the effect of vinculum of LHBT [5, 15]. Excursion testing in a previous study showed that the vinculum prevents the biceps origin from migrating distal to the groove entrance [15], and the authors claimed that pullout strength is more affected by innate strength of the vinculum than biceps associated pathologies. Furthermore, pullout tension increases with time after tenotomy, because adhesion and autotenodesis occur in the bicipital groove [7, 37, 38]. However, the maintenance of length-tension relationships is another hitherto noninvestigated aspect of tenotomy and tenodesis.

## 5. Limitations

This study has several limitations. First, pullout tension was measured by hooking the LHBT and the applied displacing force was anterior rather than inferior (the vector of biceps muscle), and, thus, measured tensions may have been different, had a direct inferior force been applied. However, friction between the LHBT and the tensiometer hook was low since the biceps tendon is surrounded by a synovial sheath, and, thus, we cautiously assumed that the friction effect would be negligible. Second, no normal control data was obtained, that is, measures of LHBT pullout tension in the absence of an adjacent tissue pathology. Furthermore, the subjects of the present study with a rotator cuff tear were somewhat aged (range: 44 to 82), because biceps tenodesis is rare in clinical practice in younger individuals, since biceps tendinitis and SLAP lesions are usually treated conservatively. Third, hidden lesions were not considered in the present study as decisions regarding tenodesis were made based on arthroscopic findings, and hidden lesions could not be evaluated during tenotomy. Accordingly, if the presence of a hidden lesion is related to status of the vinculum at the bicipital groove, pullout tensions might be affected. Lastly, for subgroup analysis in female, it is also shown that there is no statistical significance in the pullout tension according to different pathologies. However, relevant numbers in some variables were small (i.e., biceps tear and subscapularis tear); therefore, statistical result cannot be made for especially subscapularis tear in female patients.

## 6. Conclusions

The present study shows pathologies of the LHBT or of adjacent tissues do not affect LHBT pullout tensions or distal migration of tenotomized LHBTs. In fact, gender was the only factor found to affect LHBT pullout strength. Accordingly, risk of distal migration of tenotomized LHBT could not be predicted with intraoperative arthroscopic pathologic findings.

## Figures and Tables

**Table 1:** Patient data.

Age (yr)	60.4 ± 6.9 (range: 44 to 82)
Sex (M : F)	46 : 24
Height (cm)	162.9 ± 8.5 (range: 144.0 to 178.3)
Weight (kg)	67.6 ± 11.0 (range: 42.8 to 97.3)
Biceps tear (no : partial tear : partial tear with hypertrophy)	17 : 31 : 22
SSC tear (no : type I : type II : type III) [20]	16 : 26 : 23 : 5
Type II SLAP lesion (no : yes)	42 : 28
Biceps location (normal : subluxation : dislocation)	36 : 31: 3
Pulley lesion (no : anterior : posterior : anterior and posterior)	5 : 5 : 26 : 34

M = male; F = female; SSC = subscapularis; SLAP = superior labrum anterior to posterior.

**Table 2:** Subgroup analysis for male patients.

	No of patients	Pullout tension (N)	P value
Biceps tear (no : partial tear : partial tear with hypertrophy)	13 : 19 : 14	85.2 ± 40.2 : 92.4 ± 36.8 : 101.7 ± 52.8	0.612
SSC tear (no : yes)	13 : 33	93.0 ± 47.8 : 93.3 ± 41.3	0.984
Type II SLAP lesion (no : yes)	26 : 20	88.2 ± 45.3 : 99.7 ± 39.1	0.372
Biceps location (normal : subluxation or dislocation)	24 : 22	97.8 ± 45.7 : 88.2 ± 39.6	0.453
Anterior pulley lesion (no : yes)	24 : 22	97.2 ± 46.9 : 88.8 ± 38.1	0.508

SSC = subscapularis; SLAP = superior labrum anterior to posterior.

**Table 3:** Subgroup analysis for female patients.

	No of patients	Pullout tension (N)	P value
Biceps hypertrophy (no : yes)	16 : 8	70.4 ± 29.3 : 80.1 ± 54.8	0.577
Type II SLAP lesion (no : yes)	16 : 8	76.8 ± 44.1 : 67.5 ± 26.0	0.590
Biceps location (normal : subluxation or dislocation)	12 : 12	66.8 ± 27.6 : 80.5 ± 47.5	0.397
Anterior pulley lesion (no : yes)	7 : 17	67.5 ± 27.9 : 76.2 ± 42.8	0.924

SSC = subscapularis; SLAP = superior labrum anterior to posterior.
